# Early Struggles—The Relationship of Psychopathology and Development in Early Childhood

**DOI:** 10.3390/children12030265

**Published:** 2025-02-21

**Authors:** Annick Martin, Mirijam-Griseldis Galeris, Mona K. Theil, Silvano Sele, Marialuisa Cavelti, Jan Keil, Michael Kaess, Georg G. von Polier, Franziska Schlensog-Schuster

**Affiliations:** 1 Department of Child and Adolescent Psychiatry, Psychotherapy and Psychosomatics, University of Leipzig, 04103 Leipzig, Germany; mirijam-griseldis.galeris@medizin.uni-leipzig.de (M.-G.G.); mona.theil@medizin.uni-leipzig.de (M.K.T.); jan.keil@medizin.uni-leipzig.de (J.K.); georg.vonpolier@medizin.uni-leipzig.de (G.G.v.P.); franziska.schlensogschuster@upd.ch (F.S.-S.); 2 Developmental and Educational Psychology, Institute of Psychology, Friedrich-Alexander University Erlangen-Nürnberg, 91052 Nürnberg, Germany; 3 University Hospital of Child and Adolescent Psychiatry and Psychotherapy, University of Bern, 3000 Bern, Switzerland; silvano.sele@unibe.ch (S.S.); marialuisa.cavelti@unibe.ch (M.C.); michael.kaess@upd.ch (M.K.); 4 Department of Child and Adolescent Psychiatry, University Hospital Heidelberg, 69115 Heidelberg, Germany; 5 Department of Child and Adolescent Psychiatry, Psychosomatics and Psychotherapy, Medical Faculty, RWTH Aachen University, 52074 Aachen, Germany; 6 Research Centre Jülich, Institute of Neuroscience and Medicine, Brain & Behavior (INM-7), 52428 Jülich, Germany

**Keywords:** infant mental health, child development, psychological symptoms, regulatory disorder, regulatory symptoms, developmental delay

## Abstract

Background/Objectives: Early childhood psychopathology has a profoundly negative impact on various areas of psychosocial functioning. Psychopathology and child development are closely linked and influenced by a range of factors, such as socioeconomic status and pre- and postnatal risks. This cross-sectional study aims to gain a deeper understanding of child development in children with early psychopathology and to derive implications for the diagnosis and treatment of the youngest children. Methods: This cross-sectional study examines the developmental status of children aged 0 to 5 years with early psychopathology (EPP) in comparison to gender- and age-matched healthy controls (HC). A newly self-developed objective, semi-structured, symptom-based interview was administered in each subgroup by trained research assistants. This interview is based on the DC: 0–5 classification system. The ET 6-6-R was used as a standardized developmental test, covering the developmental areas of gross and fine motor skills, cognition, language development, and socioemotional development. Demographic characteristics, including maternal education and household income, were considered as potential confounders. Results: Children with early pathology elicited a lower total developmental quotient than healthy controls. HC demonstrated a better performance in fine motor skills, language development, and socioemotional development than their counterparts with EPP. HC showed better gross motor skills as well, but statistical significance was *p* = 0.08. After controlling for maternal education, overall development and socioemotional development were found to be lower in the EPP group than in the HC group. Conclusions: These findings highlight the need to identify psychopathology and associated developmental deficits early in childhood which might allow more targeted treatments, enhancing developmental opportunities for affected children.

## 1. Introduction

Mental disorders are prevalent among children and adolescents, with their onset frequently occurring during early childhood. However, particularly at this early age, mental problems are underdiagnosed or only broadly categorized as ‘regulatory problems’. This absence of thorough diagnostic evaluation in early childhood impedes the initiation of timely and effective interventions [1]. In fact, a large epidemiological study points to 16–18% of children with early mental disorders. Specifically, in children aged 0–3 years, these are mostly conduct and emotional disorders, eating disorders, and neurodevelopmental disorders [2,3].

In clinical practice, children with early psychopathology often present with symptoms described under the umbrella term ‘regulatory problems’. This term refers to sleep disturbances, feeding problems, and excessive crying [4], all of which are highly prevalent symptoms in early childhood [5,6,7]. It is well documented that the presence of early regulatory problems has an impact on a child’s development. They are closely linked and influenced by various factors, including socioeconomic status (SES) or pre- and postnatal risks [1,2]. Sidor et al. demonstrated a negative influence of infantile crying and sleep disturbances on the social development of children [8]. A study by Toffol et al. demonstrated that higher levels of infant regulatory problems predict a delayed neurobehavioral development by using the “Ages and stages Questionnaire”, a parent-completed developmental screening [9,10]. In our study, we chose to use the ET-6-6-R as an objective test with scale-specific developmental quotients, calculated from raw scores according to the ET 6-6-R manual. These were used to calculate an overall development quotient with the aim of comparing the healthy and sick groups. Further research pointed out that the vast majority (up to 95%) of children exhibiting moderate to severe regulatory problems at the age of 7 months displayed developmental delay or parent–child relationship issues at the age of 30 months. Moreover, internalizing symptoms persisted from preschool to middle school [11]. In their longitudinal study of a birth cohort, Caspi et al. demonstrated that children exhibiting impulsive, restless, and distractible behaviors at the age of three years were more likely to meet the diagnostic criteria for antisocial personality disorder or engage in delinquent behavior by age 21. Conversely, those who presented as shy, anxious, or sad at the age of three years had a higher likelihood of meeting the diagnostic criteria for depression at the age of 21. These findings highlight the long-term implications of early behavioral traits on mental health outcomes [12].

Much less research has focused on the cross-sectional relationship between early psychopathology and co-occurring developmental delays. This relationship seems complex and multifaceted: the development of a child in the first five years of life undergoes rapid changes, and parenthood has a high impact on child behavior, particularly at this age [13]. Multiple studies point to important and independent associations of both parenthood and biological factors which affect early psychopathology. Maternal anxiety disorders or family adversity and psychosocial stress factors have been shown to be associated with excessive infant crying and feeding problems [14]. Other research focusing on biological processes has shown that very preterm birth is predictive of multiple regulatory problems or of single feeding disorders [15]. The neurobiology of early childhood refers to the critical development of the brain and nervous system during the first years of life, with early experiences having a significant impact on cognitive, emotional, and social abilities. During this period, the brain is highly plastic and sensitive to environmental influences, with the quality of relationships and level of stress playing an important role [16,17]. Healthy attachments and a supportive environment can promote brain development, while adverse environmental factors can have long-lasting negative effects [18]. Therefore, a bio-psychosocial understanding of a child is crucial. In this vein, the Zero to Three working group for the diagnosis of early psychopathology has added a relationship classification to the dimensions of the Multiaxial Classification Scheme for Mental Disorders of Childhood and Adolescence (MAS) according to ICD-10 [19].

To shed more light on the actual association between early psychopathology—presented as ‘regulatory problems’—and social and biological factors such as developmental delays, we aim to address the following questions: (1) which clinical diagnoses are presented as ‘regulatory problems’ according to DC:0-5^TM^? (2) Do children with early psychopathology show more developmental delays compared to a healthy control group, and which domains are mostly affected?

A better understanding of early psychopathology, along with social and developmental aspects, may add to a detailed and accurate diagnostic process. This is crucial for providing appropriate treatment and preventing ongoing mental and developmental dysfunction, which is challenging to address later on. To achieve this, we employ a cross-sectional study design examining a sample of infant and toddler outpatients at a child and adolescent psychiatry clinic.

### 1.1. Study Design

Initially, this monocentric case–control study was embedded in a multicenter project that included a cohort study and two randomized controlled trials (SKKIPPI; [20,21,22]), funded by the German Health Care Innovation Fund. To increase the number of the participants, recruitment was continued as part of a second study focusing on the evaluation of a semi-structured clinical parental interview in German based on the DC:0-5^TM^ classification system [23]. Inclusion criteria were sufficient German language skills of the mother in relation to productive language and comprehension, absence of severe acute psychiatric illnesses such as suicidal tendencies, psychosis, or substance abuse of the mother, serious physical illnesses of the child, and the child’s age being 60 months or younger. All legal guardians provided written informed consent.

### 1.2. Participants

We recruited two subsamples. One subsample, “early psychopathology” (EPP), consisted of *n* = 43 children and their caregivers from the special outpatient clinic for infants and toddlers. Data for this group were collected in both inpatient and outpatient settings. The second subsample consisted of *n* = 40 age-matched “healthy controls” (HC) and was recruited via public notices and flyers distributed in pediatric practices, to midwives, and in public locations such as swimming pools and kindergartens.

**Figure 1:**
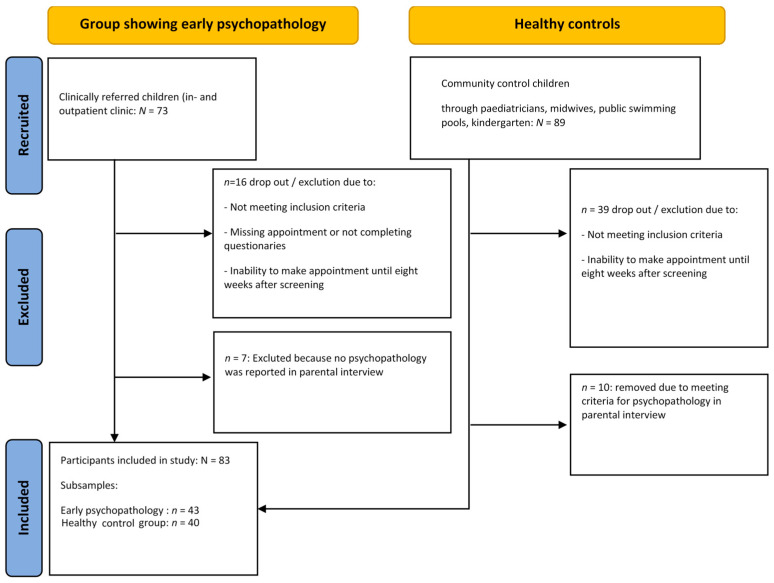
Composition of the sample.

Figure 1 demonstrates the composition of the sample, including information on recruitment, inclusion, exclusion, and drop-out rates, with an initial total of *n* = 73 patients recruited for the EPP sample and *n* = 89 families volunteering to participate as healthy controls. The final sample comprised *N* = 83 age- and gender-matched children consisting of *n*= 43 children with early psychopathology (M_Age_ = 29.9 months, SD = 18.1; 46.5% girls) and *n* = 40 children (M_Age_ = 28.1 months, SD = 15.7; 55.0% girls) in the healthy control group.

#### 1.2.1. Study Procedures

Healthy controls were screened for absence of early psychopathology. Mothers of children older than 18 months were asked to complete the Child Behavior Checklist 1½-5 (CBCL 1½-5 [24]). Children were included in the healthy control group if they scored within the normal range on the syndrome scales of the CBCL 1½-5 according to standardized scoring instructions. In the absence of infant symptom questionnaires, we chose the Infant Characteristics Questionnaire (ICQ, 28) as a screening tool for participants below 18 months of age. Although the ICQ represents a measure of child temperament, we used it as a proxy indicator of psychopathology in this age group. In view of the absence of established norms, children were excluded from the study if their total score was two standard deviations below the mean. Children of the two groups were matched for age and sex.

Mothers were invited to an appointment for a semi-structured clinical interview based on the DC:0-5^TM^ classification system lasting one hour [23,25]. The child was invited for a separate appointment with a caregiver in order to perform the development test ET 6-6-R [26]. All mothers were asked to complete a health questionnaire that included data on the child, such as birth weight or gestational age, and sociodemographic data on the parents, such as the mother’s educational degree or net household income. All assessments within this study were carried out within 8 weeks after the initial screening.

#### 1.2.2. Early Psychopathology

Trained master’s-level research assistants conducted a semi-structured interview based on the criteria of the DC:0-5^TM^ Axis 1 to quantify an infant’s psychiatric disorders in the following categories: 10: Neurodevelopmental Disorders (e.g., autism spectrum disorder); 20: Sensory Processing Disorders (e.g., sensory over-responsivity disorder); 30: Anxiety Disorders (e.g., separation anxiety disorder); 40: Mood Disorders (e.g., depressive disorder of early childhood); 50: Obsessive Compulsive and Related Disorders (e.g., obsessive compulsive disorder); 60: Sleep, Eating, and Crying Disorders (e.g., sleep onset disorder); 70: Trauma, Stress, and Deprivation Disorders (e.g., posttraumatic stress disorder); and 80: Relationship Disorders (relationship specific disorder of infancy/early childhood) [23,27]. At the time of data analysis, psychometric properties were not available for the self-administered interview based on the Axis 1 of the DC:0-5^TM^. Since the boundaries between typical psychological developmental phenomena and disorder-relevant psychopathology are often fluid in infancy and toddlerhood, a symptom was classified as disorder-relevant in this study if it met the criteria of Axis 1 according to the DC: 0–5 in the parental interview.

#### 1.2.3. Developmental Status

Developmental status was assessed using the “ET 6-6-R” [24,26]. The ET 6-6-R assesses body motor skills, hand motor skills, language, cognition, and social-emotional skills in children aged 6 months to 6 years. The ET 6-6-R has been cross-validated with other developmental tests, such as the Bayley Scales of Infant Development, demonstrating satisfactory internal and external validity [28]. Furthermore, language and cognition outcomes assessed by the ET 6-6-R found reliable prediction of the children’s intelligence quotients [29].

The test consists of 166 tasks and 79 parental questions, with different requirements set for 13 age ranges. The skills tested in older children build progressively on those tested in younger children. For each age group, a specific protocol, evaluation form, and parent questionnaire were used. Socioemotional development was assessed through the parent questionnaire, capturing typical social abilities such as cooperation, communication skills, and behavior in social situations, as well as emotional abilities such as emotion regulation, empathy, and self-awareness.

Developmental quotients were calculated from the raw values using age-specific normative tables. When interpreting the ET 6-6-R, it is important to note that the deviation of one standard deviation around the mean value of the test values can be evaluated as an inconspicuous range. Deviations of more than one standard deviation from the age group mean are considered to be above or below the average performance [24]. To calculate the total developmental quotient, the mean of the subscale quotients for gross motor skills, fine motor skills, cognition, language, and socioemotional development was determined. The subtest drawing was excluded due to the low sample size of participants in the age group from 42–72 months.

### 1.3. Statistical Analyses

Data analysis was carried out in three steps using SPSS 29 [30]. Figures were created using PRISMA [31]. First, group differences in the total developmental scores’ quotient and differences in the developmental subscale quotients’ subscales between the two groups were calculated using Student’s t-tests. Second, to identify potential confounders of the association between early psychopathology and development, we explored differences in maternal education and socioeconomic status between groups (i.e., early psychopathology vs. healthy controls) using the Mann–Whitney U test. In case of group differences, the variables were included in the subsequent multivariate analysis as covariates. Third, a multivariate analysis of covariance (MANCOVA) was then conducted to assess group differences in developmental domains while controlling for potential confounders.

## 2. Results

### 2.1. Participants

Detailed demographic characteristics of the final sample are presented in Table 1.

### 2.2. Early Psychopathology

**Figure 2:**
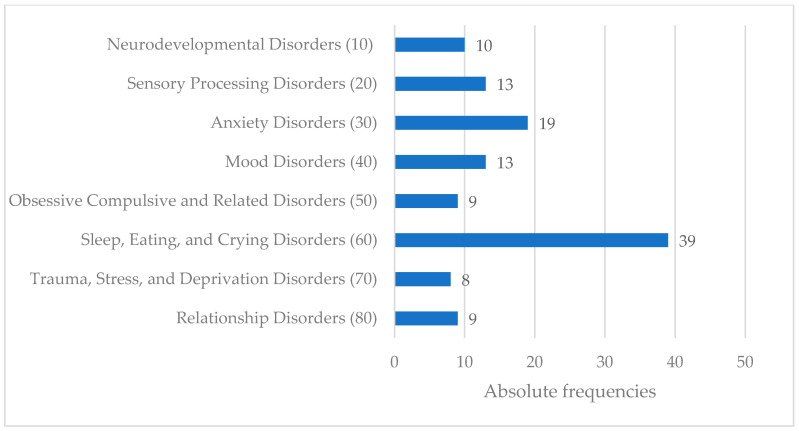
Diagnoses according to the DC:0-5^TM^ in the EPP group. The numbers in brackets refer to DC:0-5^TM^ numerical codes.

The occurrence of the various diagnoses in the EPP group according to DC:0-5^TM^ is shown in Figure 2. In the parent-based interview, 33 of the 40 children met the criteria for two or more disorders, with an average of 4.1 disorders (ranging from 1 to 13).

### 2.3. Child Development

#### 2.3.1. Total Development Quotient

**Figure 3:**
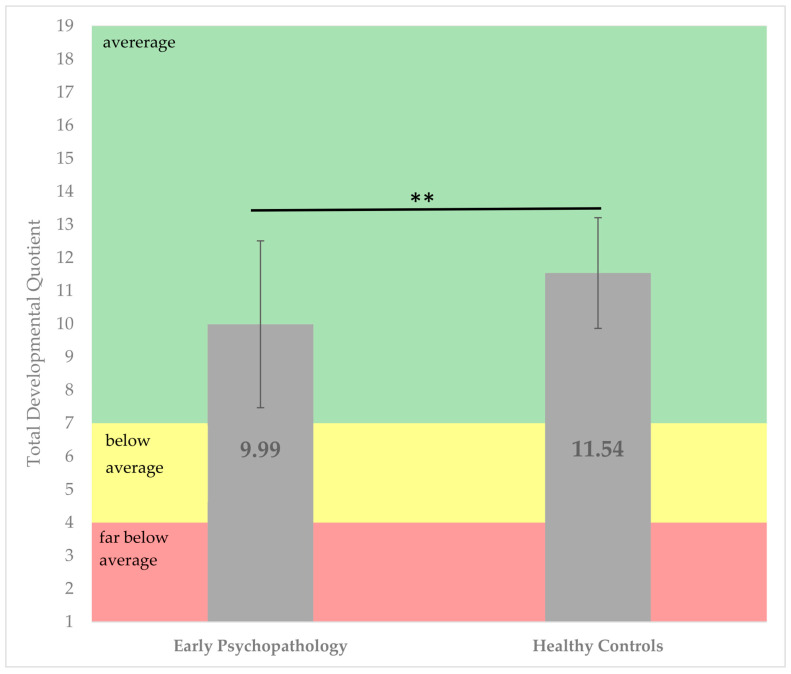
Comparison of an overall development quotient of the ET 6-6-R between children with early psychopathology and healthy controls. ** p < 0.01.

Results indicate that children with early psychopathology experienced a more pronounced developmental delay compared to children in the healthy control group. The overall developmental quotient of the healthy control group was 1.55 points higher than that of the subgroup with early psychopathology (95% CI [3.07, 12.41], *t* (80) = 3.24, *p* = 0.002; see Figure 3. Although both groups displayed developmental quotients within the normal range, the early psychopathology group elicited significantly lower scores.

#### 2.3.2. Developmental Domains

Healthy controls demonstrated higher developmental quotients in fine motor skills (*p* = 0.023), cognition (*p* = 0.031), language development (*p* = 0.007), and socioemotional development (*p* ≤ 0.001) than their counterparts with early psychopathology. Notably, no significant difference but a trend was found in the domain of gross motor skills (see Table 2).

#### 2.3.3. Analysis of Confounding Factors

A Mann–Whitney U test was calculated to determine differences in maternal education between the samples. There was a statistically significant difference in maternal education between the groups (*U* = 577.000, *Z* = −2.794, *p* = 0.005). No group differences were found for the variables sex (*t* (81) = −0.485, *p* = 0.629), age (χ^2^ (1) = 0.597, *p* = 0.440), and net household income (*U* = 714.500, *Z* = −1.230, *p* = 0.219).

#### 2.3.4. Influence of Confounding Factors

A MANCOVA was conducted to examine the difference between the two groups with respect to participants’ scores in development, while controlling for maternal education. The multivariate tests indicated a significant group difference, but no effect of maternal education (Wilks’ Lambda = 0.81, F (3.46) = 5.00, *p*= 0.007, partial η^2^ = 0.19). In bivariate analyses controlling for maternal education, fine motor skills, cognition, and language development showed moderate effect sizes (d ≈ 0.40) but *p*-values of or above 0.05 (Table 3). In contrast, socioemotional skills exhibited a large effect size (d = 0.94) with strong statistical significance (*p* ≤ 0.001).

## 3. Discussion

The present study investigates differences in developmental status between children with early psychopathology and age- and gender-matched healthy community controls using objective and standardized psychopathology and developmental assessments. Difficulties with feeding, crying, and sleeping were the most common reasons for presentation in the clinical sample “early psychopathology” (EPP). Additionally, the co-occurrence of several regulatory problems could be observed. Both of these findings have been consistently reported in population-based studies, thereby validating the comparability of our sample, characterized by early psychopathology, with cohorts from existing literature [2,32].

The main finding of this study highlights an overall lower developmental score in the group with early psychopathology compared to the healthy control group. Looking at the developmental subscales, the groups show differences in the areas of fine motor skills, cognition, language development, and socioemotional development. This observation clearly indicates a connection between psychopathology and developmental delay in early childhood. It strongly supports the introduction of standardized diagnostic procedures to assess psychopathology in the youngest patients, especially when they first present with psychiatric symptoms, such as regulatory disorders, to their primary caregivers. Our results are in line with earlier studies: DeGagni and colleagues described that children who initially presented with regulatory problems showed deficits in the developmental areas of emotional maturity, motor coordination, and tactile sensitivity in the follow-up assessment after four years [33]. Furthermore, it has been reported that up to 80% of children with developmental delays demonstrate feeding difficulties [34]. Pant and colleagues found that children with motor development disorders are more likely to be diagnosed with a mental disorder before the age of eight [35]. Additionally, abnormal language development, neurocognitive functioning, and impaired social interactions at the age of 10 months are demonstrated predictors of psychiatric disorders at 1 ½ years [36,37]. Wolke et al. report that excessive crying and feeding problems in infancy affect cognitive development. In girls, regulatory disorders directly predicted lower cognitive functioning at 56 months of age. In boys, this effect was mediated by delayed mental development at 20 months of age [38].

By further analyzing developmental domains in our study, gross motor skill differences between groups were not statistically significant but approaching statistical significance. Earlier research points to a link between motor development delays in early childhood and increased rates of anxiety disorders and depression in later childhood [39,40]. Longitudinal studies with larger sample sizes are necessary to further clarify this relationship.

By investigating relevant confounders, we found maternal education, measured as the highest academic degree, to be lower in the group with early psychopathology. This is consistent with earlier research that describes lower maternal education and a migration background as increasing the risk of combined regulatory problems [32,41]. In contrast, net household income was not identified as a confounding factor in this study. This is somewhat surprising, given that other studies have consistently shown that a lower social status is associated with a higher prevalence of mental health problems in children [42]. This discrepancy with the literature might be related to the sample size of this study.

After controlling all analyses for maternal education, maternal education was not associated with overall development or developmental domains. Early psychopathology remained associated with an overall lower development and a lower socioemotional development. Notable trends with medium effect sizes but no significant results were observed in relation to language development, cognition, and fine motor skills. This finding suggests that a portion of the observed variance may be attributable to maternal education, as it has been documented in previous studies, but this was not significant in our data. When interpreting the results for socioemotional development, it is important to consider that this scale of the ET 6-6-R relies on parental reports rather than clinician-based evaluations.

The association between early psychopathology and developmental delay is in line with other studies [37,43,44,45]. It suggests that early psychopathology may have an impact on various developmental domains. This, in turn, could further complicate the resolution of subsequent developmental tasks and exponentially increase developmental risk. The extent to which this developmental delay in young children might be reversible cannot be determined from the present data. However, we found that very young children who present with regulatory problems already show developmental delay.

Given the necessity of understanding and treating a child’s symptoms in a bio-psycho-social context, it is of great importance to obtain a comprehensive personal and family history encompassing psychosocial and somatic–biological factors in addition to the physical examination. Our findings highlight the crucial need for the early integration of standardized assessments to evaluate psychopathology and potential developmental delays in infants and toddlers. The treatment of early childhood disorders is typically conducted by an interdisciplinary team. Further studies are required to investigate the efficacy and indications of the individual therapy components in greater depth. This will enable their targeted utilization. Developmental testing may prove a valuable outcome parameter in this respect.

One of the key strengths of our study is the comparison between a clinical group and a healthy control group, an approach which enhances the validity of our findings. Additionally, we employed a comprehensive developmental assessment, which was administered by independent investigators, ensuring objectivity and rigor in the evaluation process.

Despite these strengths, there are several limitations to consider. Given the cross-sectional design of the study, it was not possible to establish causal relationships between developmental deficits and psychopathology. Furthermore, due to the unavailability of a valid age-independent screening instrument for child psychopathology, we had to rely on multiple screening tools. This introduced the possibility that the healthy control group may have consisted of heterogeneous subgroups, potentially affecting the consistency of the results. Another limitation is the inability to form disorder-specific subgroups within the clinical group, given the sample size. To address this issue in future studies, larger sample sizes per group would be necessary. Additionally, the inclusion of a single, validated screening instrument applicable across the entire age range of the healthy control group would improve the consistency and comparability of the results.

Finally, future research should consider including an assessment of the parent–child relationship, as previous studies have shown that children who experience emotionally unresponsive caregiving tend to exhibit significant deficits across various developmental domains. This aspect is crucial for a more comprehensive understanding of child psychopathology [46]. Further studies employing larger sample sizes would be advantageous for the analysis of additional subgroups, such as premature infants or children with underlying medical conditions.

## 4. Conclusions

The study of mental disorders in infancy and toddlerhood is particularly complex due to the numerous factors influencing family dynamics and child development. Additionally, the rapid pace of development and physiological occurrence of temporary, crisis-like phases during this period complicates the distinction between normal developmental fluctuations and pathological abnormalities. These challenges highlight the need for further research in the field of early childhood psychopathology. Our data show that children with early psychopathology exhibit deviations in various areas of development. A standardized and comprehensive diagnostic process is, therefore, essential for understanding early childhood psychopathology and for accurately assessing developmental deficits. From both a clinical and a research perspective, early assessments of developmental status in children with psychiatric disorders are of central importance. Based on the identified developmental deficits, targeted therapeutic interventions can be initiated, with their effectiveness monitored through continuous evaluations of developmental progress throughout the course of treatment.

## Figures and Tables

**Table 1:** Demographic characteristics of the sample.

	Full Sample (*N* = 83)	Early Psychopathology (*n* = 43)	Healthy Controls (*n* = 40)
Child Characteristics			
Age in month (*M*, *SD*)	29.0 (16.9)	29.9 (18.1)	28.1 (15.7)
Sex (*n*, %)			
Male	41 (49.4)	23 (53.5)	18 (45.0)
Birthweight in grams (*M*, *SD*)	3345.11 (577.8)	3314.6 (582.0)	3377.9 (577.7)
Gestational age in weeks (*M*, *SD*)	39.4 (2.4)	39.33 (2.1)	39.40 (2.6)
Number of siblings (*n*, %)			
No siblings	35 (42.7)	20 (47.6)	15 (37.5)
One sibling	39 (47.6)	16 (38.1)	23 (57.5)
≥2 siblings	8 (9.8)	6 (14.3)	2 (5.0)
Maternal Characteristics			
Age in years (*M*, *SD*)	34.46 (4.4)	34.53 (4.6)	34.81 (4.2)
Origin (*n*, %)			
German	82 (98.8)	42 (97.7)	40 (100.0)
Education (median)	University degree	High school diploma	University degree
Partnered (*n*, %)	73 (90.1)	34 (81.0)	39 (97.5)
Custody of child (*n*, %)			
Shared	68 (84.0)	30 (73.2)	38 (95.0)
Mother alone	13 (16.0)	11 (26.8)	2 (5.0)
Monthly household income (median)	>3.500€	>3000€–<3500€	>3.500€

**Table 2:** *t*-Test for comparison of the means of the developmental quotients between the early psychopathology group and the healthy controls.

Developmental Quotient (DQ)	EPP	HC	*t* (df)	* p *
Gross motor skills (*M*, *SD*)	10.3 (3.9)	11.4 (2.9)	1.4 (80)	0.078
Fine motor skills (*M*, *SD*)	10.2 (3.0)	11.4 (2.4)	2.0 (80)	0.023 *
Cognition (*M*, *SD*)	10.2 (3.6)	11.7 (3.3)	1.9 (80)	0.031 *
Language development (*M*, *SD*)	10.3 (2.9)	11.6 (1.8)	2.5 (80)	0.007 **

* *p* < 0.05; ** *p* ≤ 0.001.

**Table 3:** Differences between EPP and HC using bivariate analyses controlled for maternal education.

Developmental Quotient (DQ)	Corrected *R*²	*p*	*d*
Gross motor skill	0.001	0.206	0.285
Fine motor skill	0.026	0.081	0.396
Cognition	0.019	0.072	0.408
Language development	0.070	0.056	0.434
Socioemotional	0.168	≤0.001	0.943

## Data Availability

The data presented in this study are available upon reasonable request from the corresponding author. The data are not publicly available due to the private details of the participants that they contain.
